# Advances in the understanding and management of T-cell prolymphocytic leukemia

**DOI:** 10.18632/oncotarget.22272

**Published:** 2017-11-01

**Authors:** Kamel Laribi, Pierre Lemaire, Jeremy Sandrini, Alix Baugier de Materre

**Affiliations:** ^1^ Department of Hematology, Centre Hospitalier du Mans, Le Mans, France; ^2^ Laboratory of Biology and Hematology, Centre Hospitalier du Mans, Le Mans, France; ^3^ Laboratory of Anatomopathology, Centre Hospitalier du Mans, Le Mans, France; ^4^ Department of Medicine, Clinique du Pré, Le Mans, France

**Keywords:** T-cell prolymphocytic leukemia, morphology, cytogenetic, molecular biology, treatment

## Abstract

T-prolymphocytic leukemia (T-PLL) is a rare T-cell neoplasm with an aggressive clinical course. Leukemic T-cells exhibit a post-thymic T-cell phenotype (Tdt^−^, CD1a^−^, CD5^+^, CD2^+^ and CD7^+^) and are generally CD4^+^/CD8^−^, but CD4^+^/CD8^+^ or CD8^+^/CD4^−^ T-PLL have also been reported. The hallmark of T-PLL is the rearrangement of chromosome 14 involving genes for the subunits of the T-cell receptor (TCR) complex, leading to overexpression of the proto-oncogene TCL1. In addition, molecular analysis shows that T-PLL exhibits substantial mutational activation of the IL2RG-JAK1-JAK3-, STAT5B axis. T-PLL patients have a poor prognosis, due to a poor response to conventional chemotherapy. Monoclonal antibody therapy with antiCD52-alemtuzumab has considerably improved outcomes, but the responses to treatment are transient; hence, patients who achieve a response to therapy are considered for stem cell transplantation (SCT). This combined approach has extended the median survival to four years or more. Nevertheless, new approaches using well-tolerated therapies that target growth and survival signals are needed for most patients unable to receive intensive chemotherapy.

## INTRODUCTION

Significant progress has been made in the understanding of T-PLL through better characterization by flow cytometry and identification of genomic and molecular abnormalities involved in its pathophysiology [1–4]. The prognosis remains poor with current treatment. Targeted therapies involving growth and survival signals, such as the JAK-STAT pathway, could be effective therapeutic options

### Diagnosis by morphology and flow cytometry

**Figure 1:**
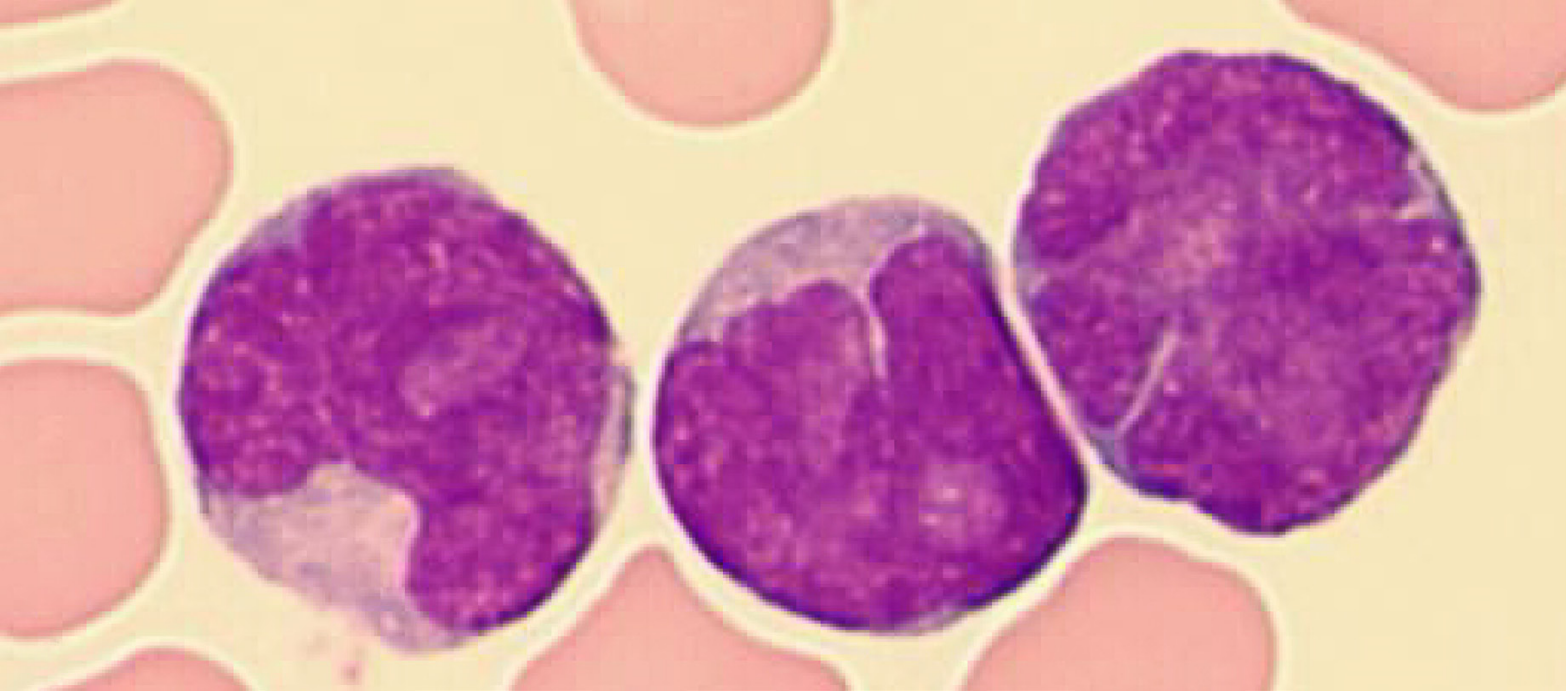
Small cells containing distinct nucleoli, irregular nuclei, and slightly basophilic cytoplasm (classical form) (MGG, X 100)

**Figure 2:**
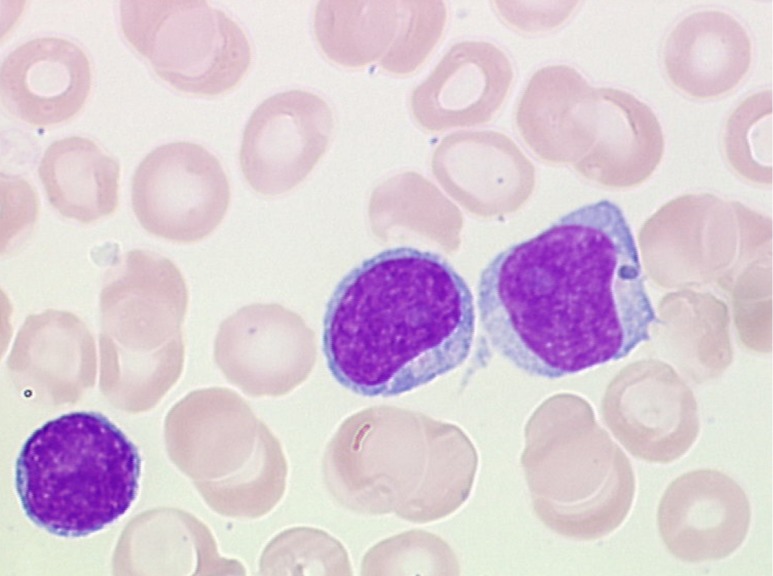
Small cell with inconspicuous nucleoli and a regular nucleus indistinguishable from CLL cells Two larger cells with distinct nucleoli, round-oval nuclei, and basophilic cytoplasm (MGG, X 100).

**Figure 3:**
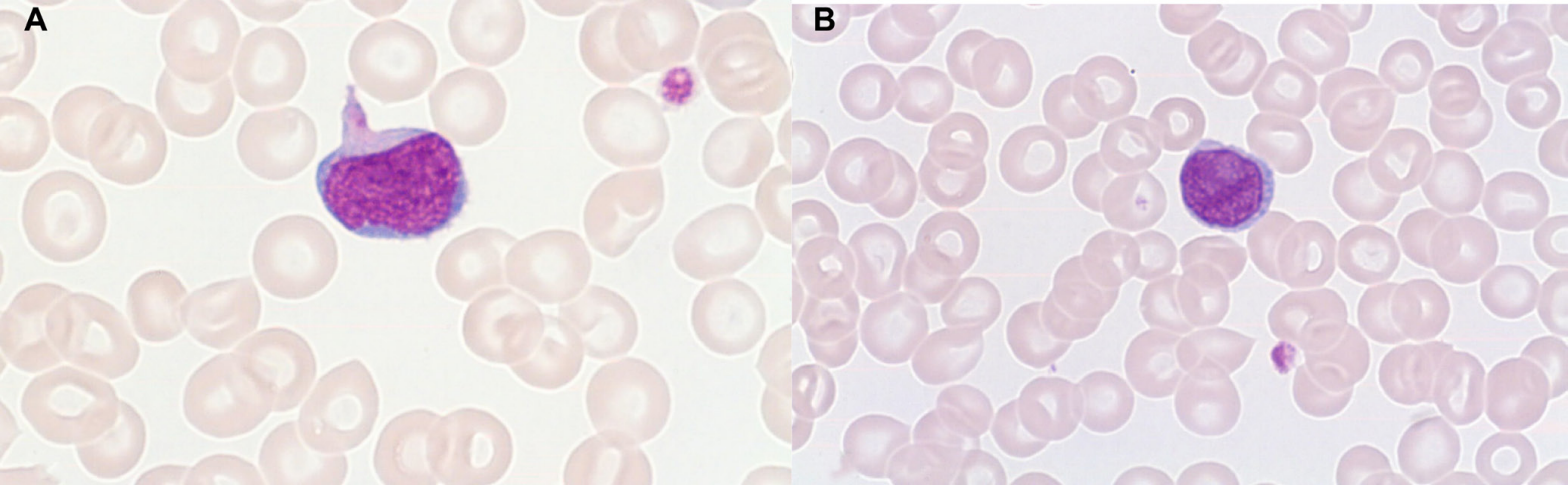
Small cell with fine chromatin, cytoplasmic extensions, inconspicuous nucleoli, and basophilic cytoplasm **(A)**. Small cell with distinct nucleoli and folded (wrinkled) nucleus **(B)**: Sezary aspect (MGG, X 100).

**Figure 4:**
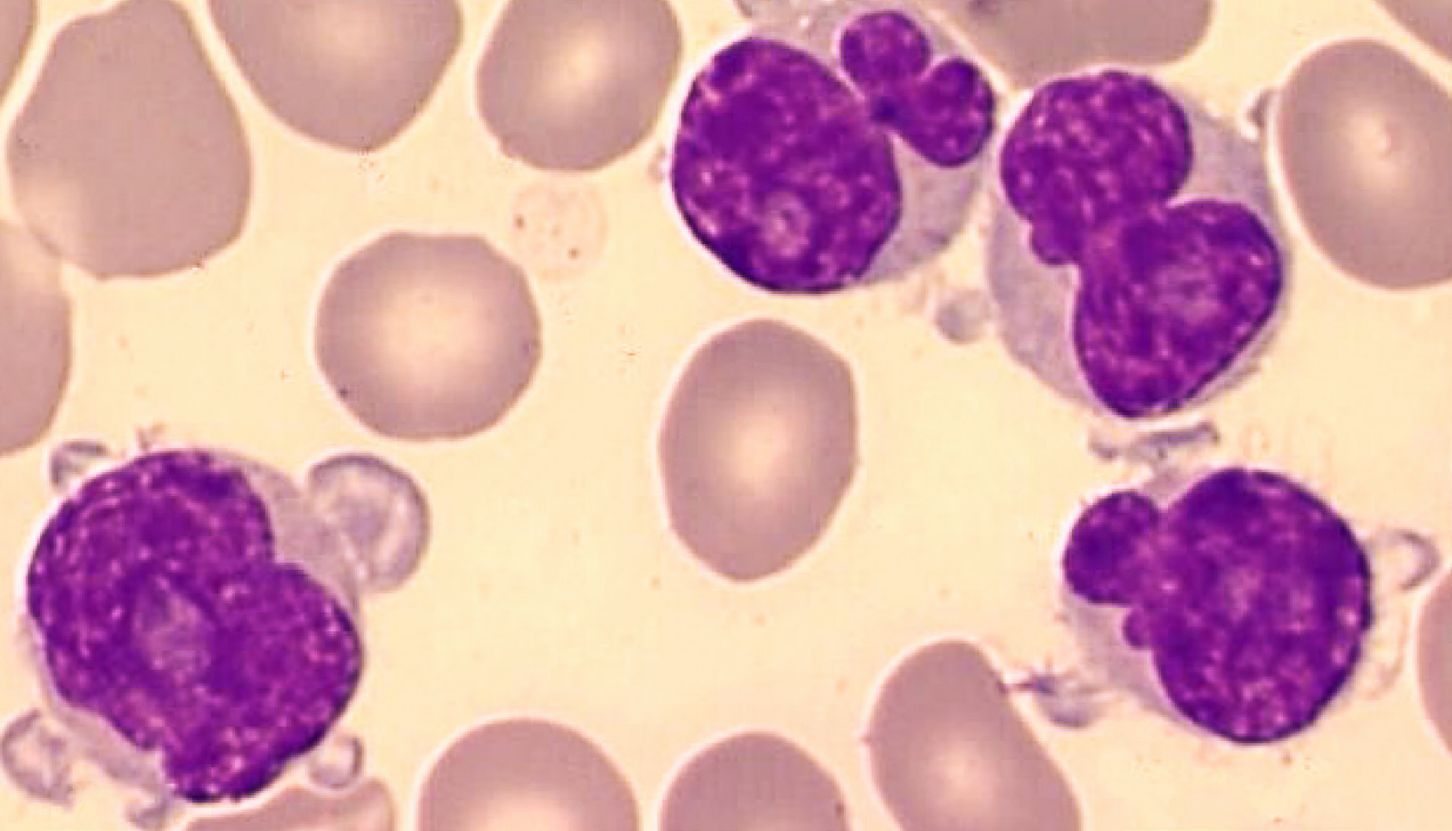
Small and medium-sized cells containing distinct nucleoli and very irregular nuclei (flower-like appearance): ATLL aspect (MGG, X 100)

**Figure 5:**
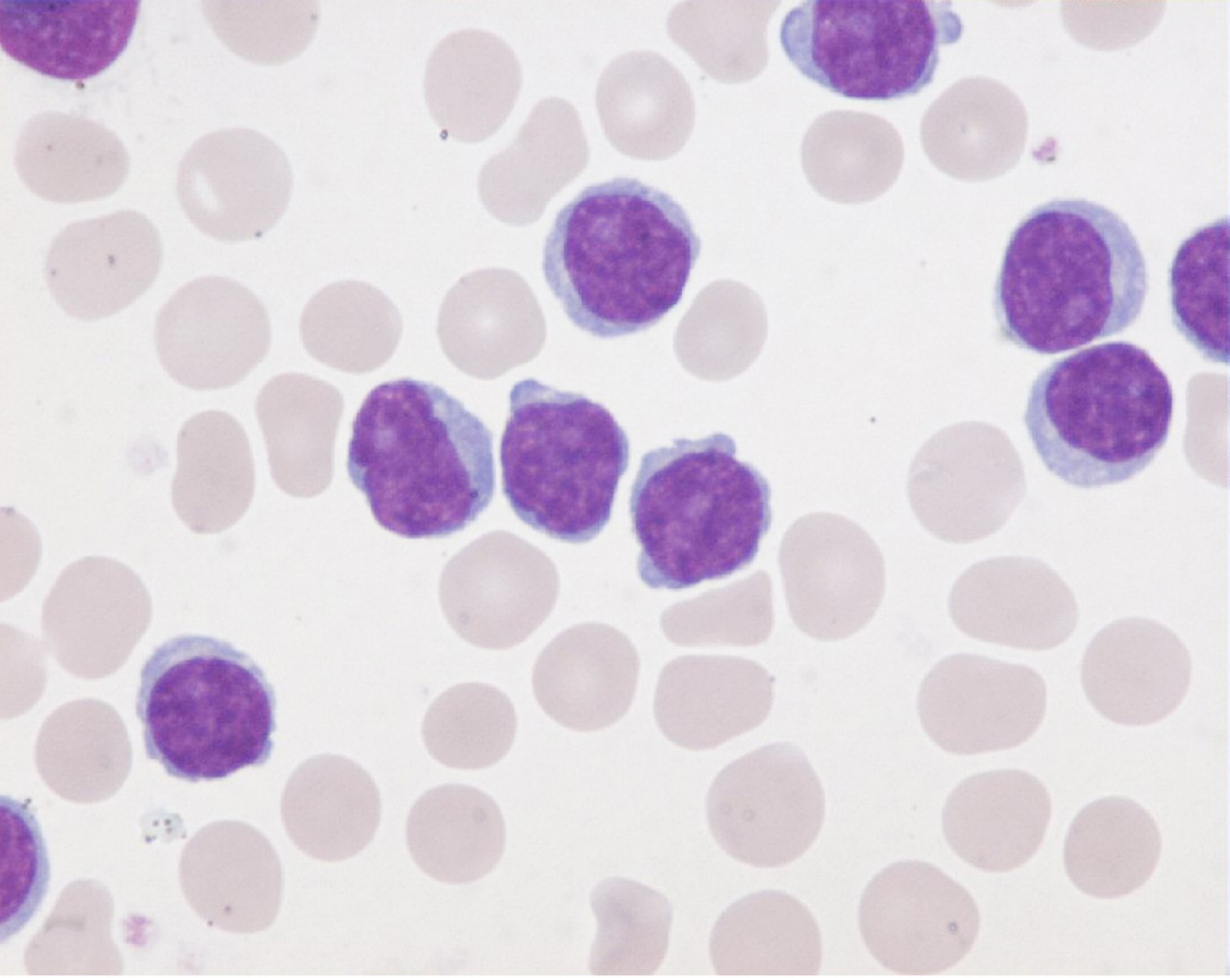
Small cells containing distinct nucleoli, basophilic cytoplasm, and irregular nuclei, barely distinguishable from CLL cells, except for the presence of characteristic cytoplasmic extensions (Blebs) (MGG, X 100)

**Figure 6:**
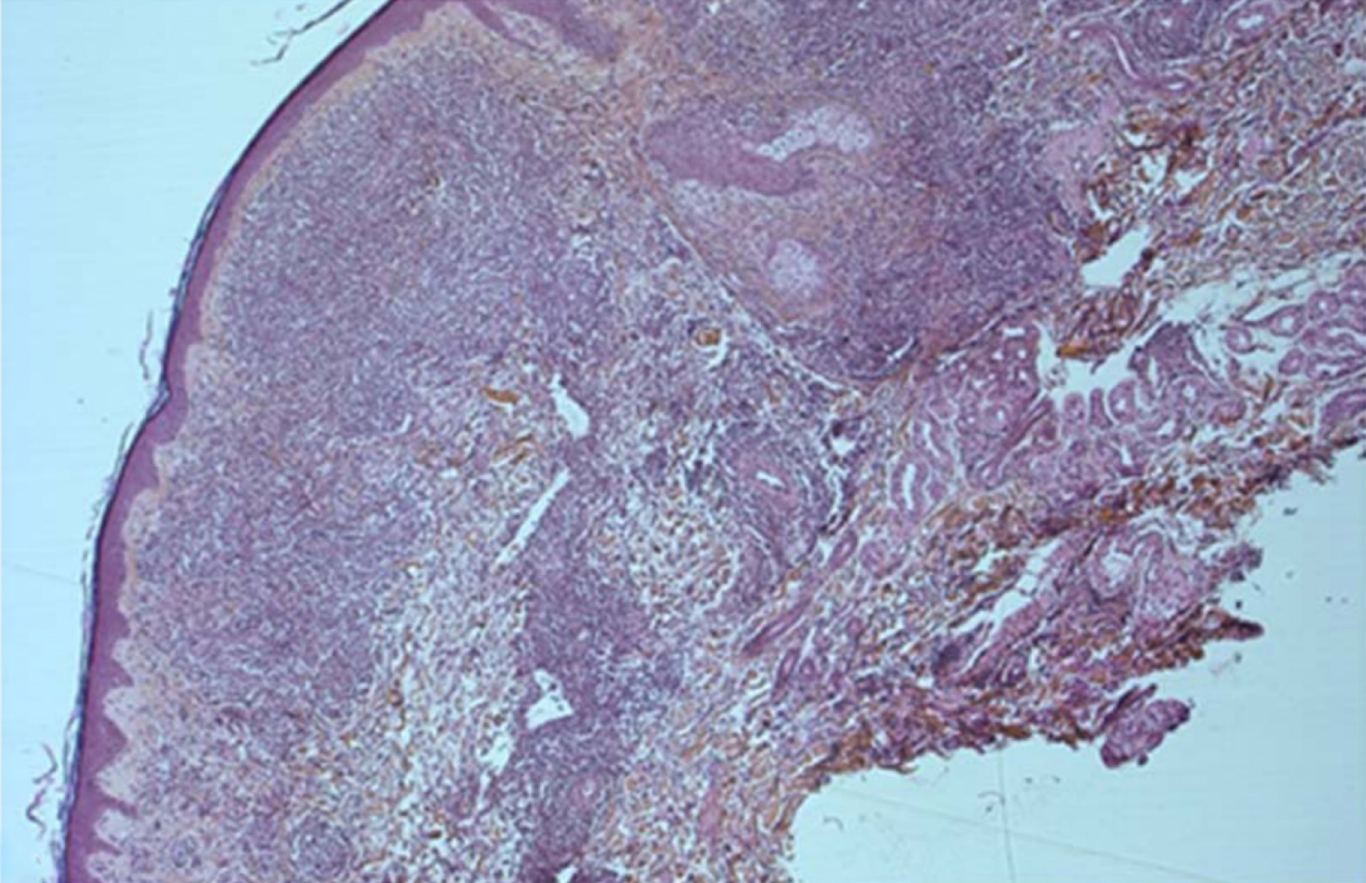
Dermal infiltration with perivascular and periadnexal involvement without epidermotropism (HES, X 5)

**Figure 7:**
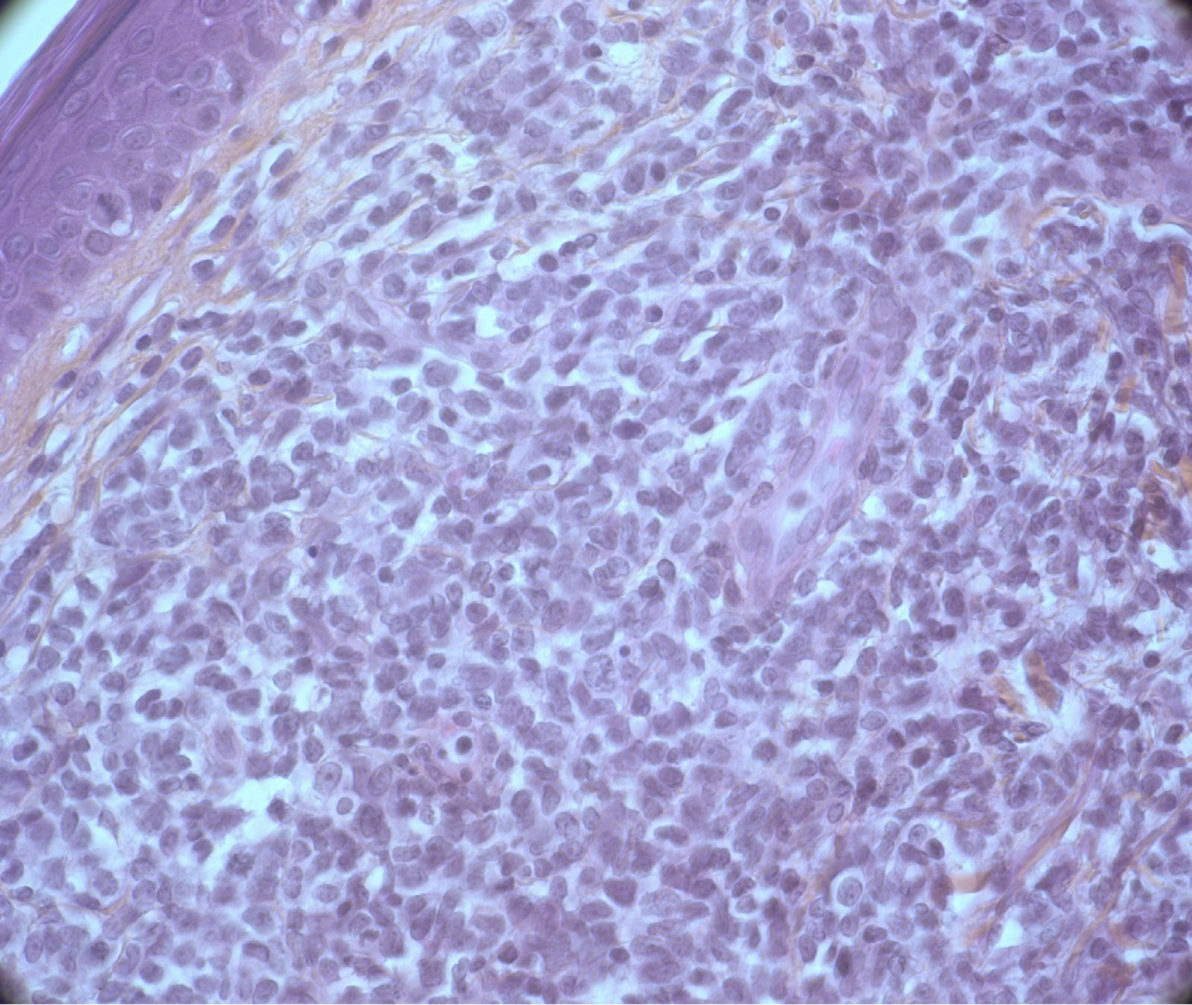
Dermal infiltration by medium-sized cells with irregular immature nuclei (HES, X 40)

**Figure 8:**
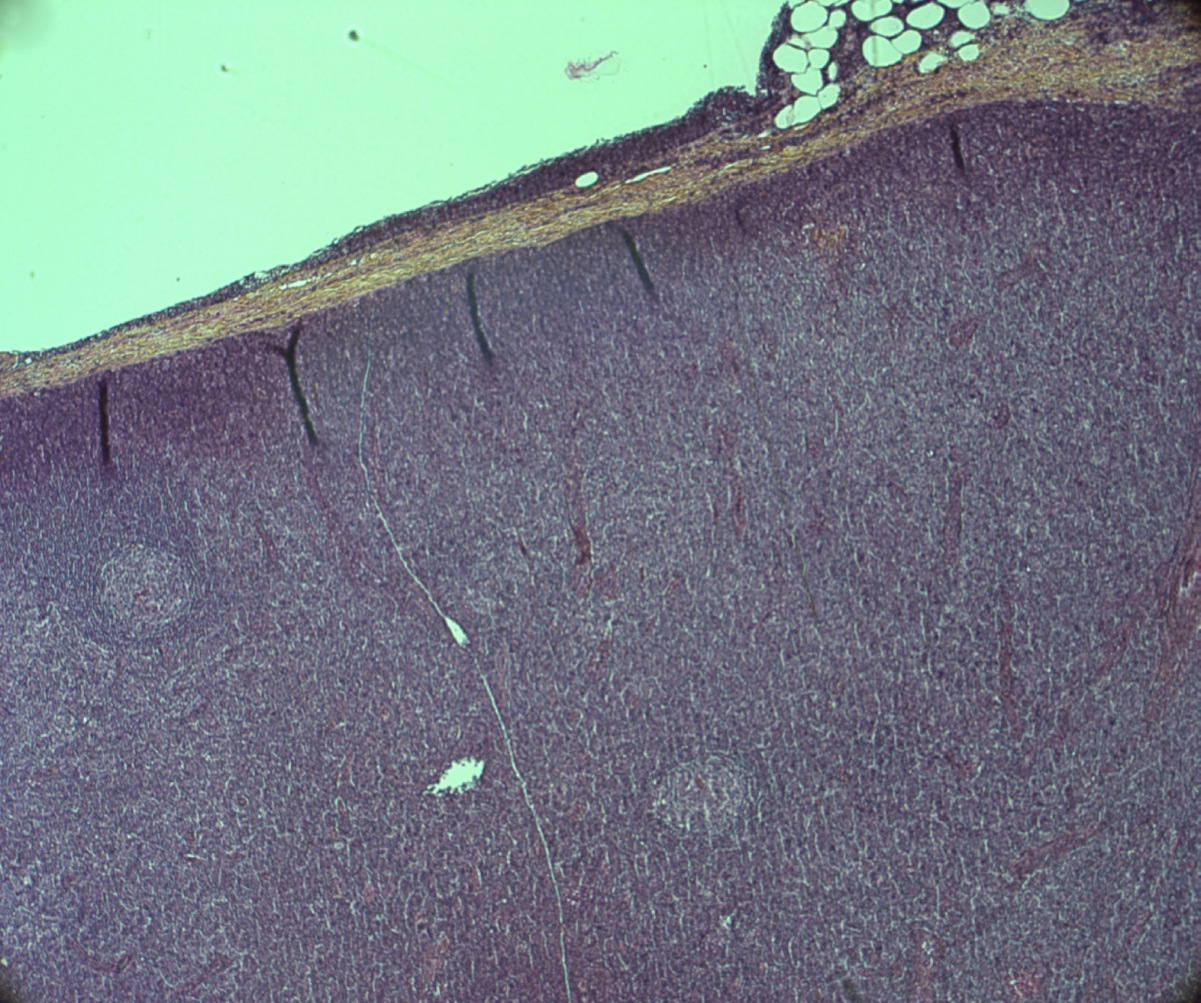
Diffuse lymph node infiltrate with paracortical expansion Persistence of two residual lymphoid follicles (HES,X 5).

**Figure 9:**
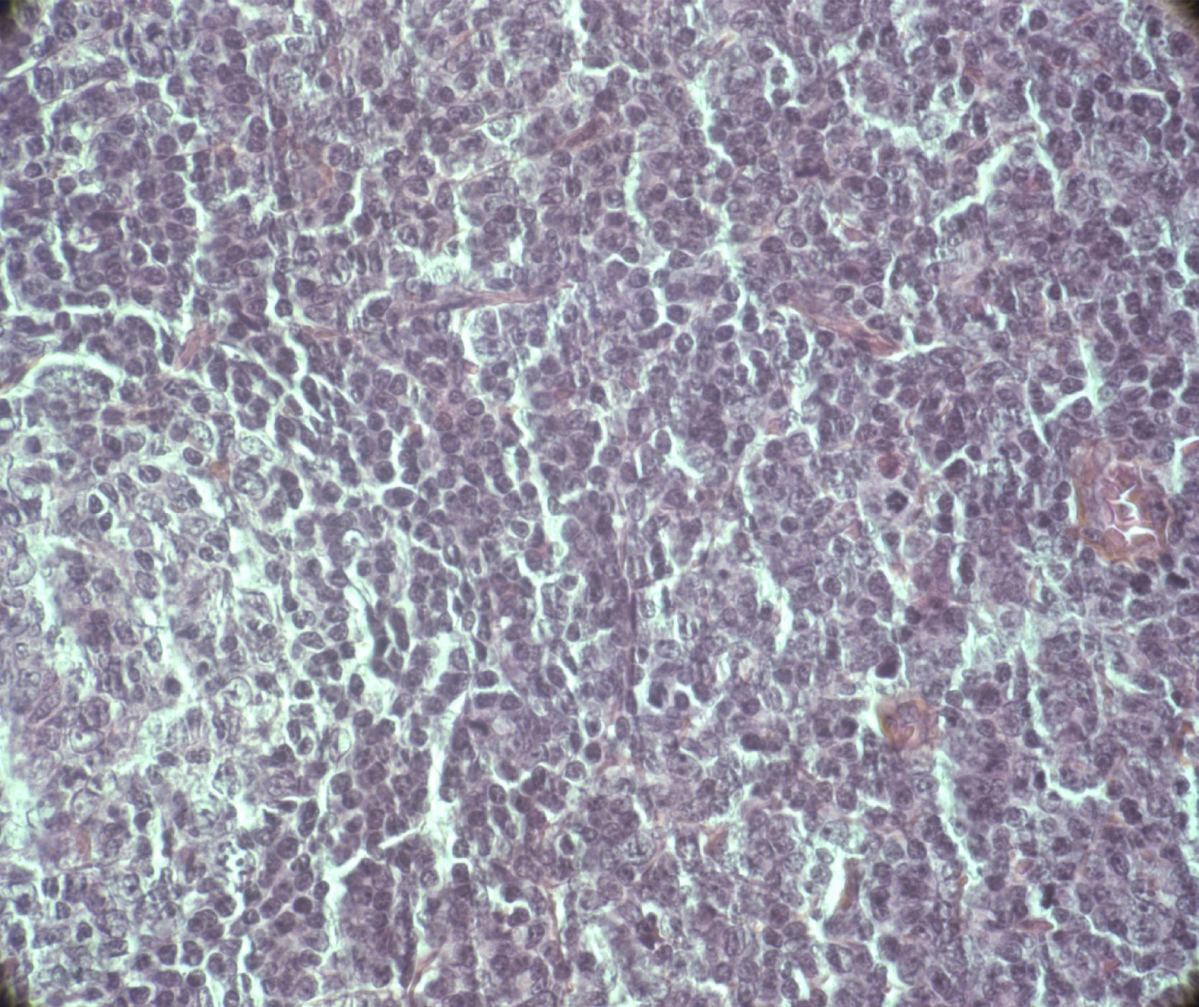
Lymph node infiltrate by medium-sized cells with irregular nuclei (HES, X 40)

T-PLLs generally have a classic prolymphocytic morphology consisting of medium-sized cells and a high nuclear/cytoplasmic ratio, condensed chromatin, basophilic cytoplasm with cytoplasmic blebs, and a single prominent nucleolus (Figures 1, 2). In half of cases, the cells contain regular, round or oval, nuclei, whereas in the rest, the nuclei are irregular and often convoluted, resembling those seen in Sezary syndrome (Figures 3A, 3B) or ATLL cells (Figure 4). In 25% of cases, the cells are small, indistinguishable from CLL cells, or cerebriform (Figures 2, 5) and the nucleolus is not easily visible by light microscopy, as it is masked by the highly-condensed chromatin [1, 5–7]. Skin lesions are characterized by leukemia cutis with perivascular and periadnexal infiltrates containing irregular, small to medium-sized lymphocytes, without epidermotropism (Figures 6–7). Infiltration of lymph nodes is often diffuse, with paracortical expansion in some cases (Figures 8–9).

By flow cytometry, T-PLL exhibits a post thymic (TdT–, CD1a–), mature T-cell immunophenotype (CD2+, CD5+, CD7+, CD16–, and CD56-) and variable CD4 and CD8 expression (Table 1). The leukemic lymphocytes are generally CD4+/CD8-, but significant subsets co-express CD4 and CD8, or are CD8+/CD4-. A CD4-/CD8- phenotype has also been reported in very rare cases. CD7 is also generally highly expressed [1, 2, 8, 9] T-PLL patients are also negative for human T-cell leukemia virus type 1.

**Table 1:** Immunophenotypic markers in T-PLL

	Matutes (1)		Chen (2)^a^		Garant (9)^b^		Garant (9)^c^	
Immunophenotype	Number of cases tested	% positive	Number of cases tested	% positive	Number of cases tested	% positive	Number of cases tested	% positive
CD1a	55	0	11	0	38	3	13	0
CD2	77	99	29	100	53	100	18	94
CD3	68	81	29	89.7	53	94	19	89
CD5	27	100	29	100	51	100	18	100
CD7	56	93	29	100	46	93	14	93
CD4+CD8−	72	65	29	37.9	53	60	19	74
CD4+CD8+	72	21	29	41.4	53	17	19	24
CD4-CD8+	72	13	29	17.2	53	15	19	0
CD4-CD8-	72	1	29	3.4	53	8	19	6
CD25	44	18	17	35.3	33	15	10	20
CD38	33	52			17	53	10	20
HLA-DR	52	8			41	0	11	9
CD10			23	0	34	3	15	0
CD16			10	0				
CD34			27	0				
CD45			29	93.1				
CD52			19	100				
CD56			29	0	30	3	11	0
TdT			22	0				
Tαβ^+^	25	60			23	96	11	82
Tγδ^+^					23	0	11	0
CD45RO^+^ CD45RA^-^					18	83	10	10
CD45RO^-^ CD45RA^+^					18	11	10	40
CD45RO^+^ CD45RA^+^					18	6	10	10
CD45RO^-^ CD45RA^-^					18	10	10	40

^a^29 population subsets analyzed among 20 cases; ^b^initially aggressive forms; ^c^initially indolent forms.

The cells of T-PLL patients are of mostly the TCR αβ phenotype, whereas only rare cases of TCR γδ expression have been reported. CD3 and TCR β may be lacking at the surface of T-PLL cells, but they are always found in the cytoplasm, and the TCR β and/or γ chain genes are rearranged in all cases [1, 8, 10].

There is generally high CD45 expression except for rare cases [2]. CD52, a glycosylphosphatidylinositol-linked protein present on normal and malignant lymphocytes and frequently targeted therapeutically in T-PLL, is expressed at high density in most T-PLL cases [11].

Markers related to T-cell activation, such as CD25, class II HLA-DR, and CD38 may or may not be expressed [1, 2, 9], whereas all T-PLL cases are negative for the T-cell Intracellular Antigen 1(TIA-1), a cytotoxic granule -associated protein expressed by cytotoxic T-cells, even in cases with a CD8+ phenotype [12].

CD117 (KIT), a type III receptor tyrosine kinase that participates in intracellular signal transduction in several cell types, is associated with the myeloid lineage, usually expressed in myeloid blasts, and aberrantly expressed on occasion in T-cell lymphoblastic leukemia (T-LL) [13], but has been rarely reported in T-PLL [2, 14].

Both cases with naïve (CD45RA+, CD45RO-) and memory (CD45RA-, CD45RO+) T-cell antigen expression have been reported [9, 15]. The memory phenotype was associated by one group with more aggressive disease [9].

The weak surface expression of CD3 and CD45, expression of both CD4 and CD8, and high CD7 expression suggest that T-cell prolymphocytes might be at an intermediate stage of differentiation between thymic and post-thymic T cells.

This phenotypic heterogeneity is further demonstrated by the report of cases with two or more distinct abnormal subsets, with distinct expression of CD4 and CD8 [2]. Furthermore, a switch from CD4 to CD8 expression during the evolution to the more aggressive form [16] has also been reported, as well as a change in the CD4/CD8 phenotype in relapsed or progressive cases relative to that at diagnosis, with increased CD8 expression and the same clonal T-cell receptor gene rearrangement at both time points [2, 16]. This may be related to clonal evolution on an oligoclonal background, infiltration by T-PLL cells initially present in other tissues, or potential therapeutic selection of preexisting undetected sub-clones at diagnosis.

However, even if certain morphological and phenotypic characteristics strongly suggest T-PLL, it is in some cases difficult to distinguish them from other mature T-leukemia. This issue is addressed later in the chapter devoted to differential diagnosis.

### Cytogenetic and molecular biology

#### Cytogenetics/array-based CGH

Classical cytogenetics have revealed the presence of complex karyotypes with recurrent chromosomal abnormalities. Genes involved in cell cycle regulation, apoptosis, and DNA repair have been identified in the transcriptomes of purified T- PLL blood samples using DNA microarray technology [3, 17–28].

Recurrent genetic alterations include translocations involving either TCL1 at 14q32.1, or MTCP1 at Xq28, inactivation of the ATM gene by deletion and/or mutation, isochromosomes of chromosome 8, and haplo-insufficiency of the CDKN1B gene.

Recurrent changes involving chromosome 14 are the most common genetic alteration, including inv(14) (q11q32) and t(14;14) (q11;q32), observed in more than two thirds of cases. These rearrangements involve the TCR α locus in 14q11 and the TCL1 locus in 14q32 resulting in the juxtaposition of these two genes and leading to activation of the proto-oncogene TCL-1 [3, 17–19, 22–25]. TCL1 plays an essential role in the pathogenesis of T-PLL, as its overexpression leads to activation of protein kinase B (Akt), resulting in increased cell proliferation [20, 25–26]. TCL1 acts as a cofactor of Akt1 that enhances Akt1 kinase activity and promotes its nuclear transport [26].

TCL6, also situated in the breakpoint cluster region on 14q32, has been suggested to be a candidate target of inv(14) (q11q32.1) [27].

Approximately 20% of patients have abnormalities involving Xq28 (MTCP-1 locus), resulting from t(X;14) (q28;q11) or t(X;7)(q28;q35). The translocation t(X;14) (q28;q11), involves rearrangement of the TCR α locus with the proto-oncogene MTCP1 (belonging to the TCL-1 gene family) [3, 21, 24], resulting in increased cell proliferation.

Abnormalities affecting chromosome 8 have been reported in more than two-thirds of cases and consist mostly of trisomy for 8q, resulting predominantly from an iso(8)(q10) and an increased expression of MYC, or rearrangements with 8p12 [3, 17–19, 22–25, 29]. 8p is known to contain various suppressor genes associated with the emergence of solid tumors [30–32], and it was suggested that the loss of a tumor suppressor gene or activation of an oncogene on 8p cooperates with increased dosage of the q arm and/or the expression of TCL-1/MTCP-1 to allow the malignant phenotype to emerge [19].

The ATM protein plays a central role in the cellular response to DNA damage. Cells with deletions/mutations of ATM are characterized by impaired apoptotic responses to DNA damaging agents. Phenotypically, ATM mutant cells exhibit impaired DNA double strand break repair [33]. Changes in 11q23 are rarely detected by cytogenetics, but molecular analysis often detects deletion and/or mutation of the ATM gene in some sporadic T-PLL cases [26, 28, 34].

In contrast to the genetic instability expected from ATM inactivation, few non-recurrent changes have been reported, but several other recurrent chromosomal abnormalities have been associated with T-PLL neoplasms: loss at 22q11, 13q, 6q, 9p, 12p, 11p11-p14, and 17p, and gains at 8q; 22q21; 14q32; 22q21–qter, and 6p [24, 35], or co-existence of t(X;14)(q28;q11), t(Y;14)(q12;q11) and a ring chromosome derivate from i(8)(q10) [36].

Some have suggested that gains of 8q result in c-MYC amplification, which directly activates the NBS gene product (NBS1 or nibrin). Overexpression of the NBS1 gene increases cell proliferation, and has a potential causal role in T-PLL progression, through the activation of the phosphatidylinositol 3-kinase/Akt signaling pathway [28, 37, 38].

### Next generation sequencing/whole exome sequencing

More recently, genomic sequencing studies of T-PLL have highlighted other events that affect the pathogenesis of T-PLL. Whole-genome sequencing (WGS) and whole-exome sequencing (WES), have revealed mutually exclusive mutations that affect IL2RG, JAK1, JAK3, or STAT5B in three quarters of cases, and identified mutations in recurrently altered genes involved in DNA repair, epigenetic transcriptional regulation, and proteasomal degradation, including EZH2, FBXW10, and CHEK2 [4].

JAK1 and JAK3 belong to the Janus kinase (JAK) family of intracellular, non-receptor tyrosine kinases that transduce cytokine-mediated signals via the JAK-STAT pathway, involved in regulating the transcription of several genes [39].

Several studies have reported a high frequency of mutations in genes of the JAK- STAT pathway with gains of functions involving JAK1, JAK3, and STAT5B, not unlike those of JAK2, TYK2, and STAT3 which have been described in other mature T-cell leukemias [15, 40, 41].

The IL2RG-JAK1-JAK3-STAT5B mutations lead to elevated transcriptional activation of STAT5, and induce constitutive STAT5 hyper-phosphorylation, as well as oncogenic transformation. Inhibition of STAT5 leads to a large reduction in cell proliferation and viability of JAK-mutated cell lines, and primary T-PLL patient samples bearing JAK-STAT pathway mutations [4].

MTCP1 TEL–JAK2 double transgenic mice have also been used to show oncogenic cooperation between MTCP1 and activation of the JAK–STAT pathway. MTCP1 and TEL–JAK2 transgenic mice were crossed and monitored for the onset of leukemia. MTCP1 TEL–JAK2 double transgenic mice died significantly earlier than mice of the other three genotypes. At 269 days, surviving mice were represented as follows: 100.0% WT, 91.0% MTCP1, 90.4% TEL–JAK2 and 6.2% MTCP1 TEL–JAK2, (*p* < 10^−9^) [39].

Mutations in JAK3 have been recently reported in 21–42% of T-PLL patients. The JAK 3^M511I^ mutant protein was found in 53–62% of cases [4, 40–42]. Another minor hotspot mutation JAK3^A573V^ was found in 12-16% of cases [4, 40]. Mutations in JAK1 have been found in 6-10% of T-PLL [4, 40, 42].

Some patients had more than one JAK mutation, suggesting that these mutations either have additional effects or independent JAK mutations occurred in different sub-clones [4, 40, 41]. Bellanger *et al.* also suggested that JAK mutations may be acquired during the evolution of the disease. One patient carried a JAK1 mutation (JAK1 c.1886_1891del) at a low level in the chromatogram, confirmed by sub-cloning. This was unexpected given the almost pure leukemic population from which the sequence was obtained [40].

Subsequent WES RNA sequencing analysis revealed somatic mutations in genes involved in DNA binding and chromatin remodeling, such as IKZF1 (N159S) and HDAC8 (I115R), or the kinase signal pathway such as NTRK1 (R33W), TNIP2 (K104Q), VAV3 (C282Y and splice site mutation), EML4 (L548W and F304S), AP2A2 (P514L), and RARB (G90W). RNA sequencing also revealed several fusion transcripts resulting in early stops of several different genes including PTPRT, L3MBTL1, and UCKL1 [15].

Next generation amplicon deep-sequencing has been performed to analyze potential mutations in the ATM, BCOR (BCL6 corepressor), and TP53 genes. The ATM gene had the highest frequency of mutations (73%), whereas the mutation frequency was much lower for the TP53 (14%) and BCOR (8%) genes [42].

The same team studied the correlation between cytogenetic aberrations and molecular mutations, and identified two distinct genetic subgroups: The first subgroup, representing 86% of cases, showed abnormalities involving the TCRA/D locus, with higher frequencies of i(8)(q10) and ATM mutations, whereas the second subgroup, including the remaining 14%, was characterized by the lack of a TCRA/D rearrangement, rare cases of i(8)(q10), and a higher frequency of TP53 mutations. The TP53 mutated subgroup was found mostly in samples from older patients (albeit not statistically significant), whereas T-PLL in younger patients was essentially characterized by translocations involving the TCRA/D locus [42].

Deletions in 12p13 that lead to haplo-insufficiency of the CDKN1B gene have been suggested to play a role in the pathogenesis of T-PLL, as this gene encodes the cyclin-dependent kinase inhibitory protein p27 (KIP1), which functions in cell-cycle regulation [43, 44].

Recently, Lopez *et al.* reported mutations in a subset of 23 T-PLL cases in the genes encoding the epigenetic regulators EZH2 in 13% (3/23), TET2 in 17% (4/23), and BCOR in 9% (2/23) of the cases [45].

Recently, Zhang et al performed whole exome sequencing and transcriptomes in a series of 12 T-PLL samples and reported mutations in NOTCH and altered of Wnt/β-Catenin pathways, indicate dysregulated embryonic developmental in the pathogenesis of T-PLL. They identified novel (KDM6A and KDM6B) and previously reported mutations (ATM, TET2) in chromatin regulatory genes, emphasizing the importance of epigenetic dysregulation in the pathogenesis of T-PLL [46].

The last several years have witnessed an acceleration of our understanding of the mechanisms involved in the pathogenesis of this disease, with the identification of novel mutations and oncogenic cooperation, which will open the way for targeted therapies.

### Clinical aspects

T-PLL is a rare disease accounting for 2% of mature lymphocytic leukemias and approximately 20% of PLL. Within mature T -cell leukemia, T- PLL may represent up to 40% of cases. This is a disease of older adults with a median age at presentation of 65 years [47], and occurs more frequently in males (male:female = 2:1) [1, 47]. Patients typically present with aggressive, widely disseminated disease at diagnosis, and a poor outcome. In a minority of cases, indolent forms can preexist at diagnosis, usually evolving to a more aggressive form [9]. In T-PLL, the white cell count is usually > 100 × 10^9^/L at presentation, as in B-PLL. Otherwise, most patients present with splenomegaly, lymphadenopathy, hepatomegaly, and skin lesions which include skin nodules, a maculopapular rash, or more rarely, erythroderma (Table 2) [1, 5, 9, 48, 49]. Peripheral edema, particularly periorbital and/or conjunctival, occurs relatively frequently and seems to be particularly characteristic of T-PLL. Pleural or peritoneal effusions also sometimes occur. Other extra-nodal sites [50], which may be seen at presentation or during evolution, include the central nervous system (CNS).

**Table 2:** Clinical characteristics of T-PLL in previously reported series

	Matutes (1)	Matutes (5)	Garand (9)^a^	Garand (9)^b^	Ravandy (48)
Number of patients	78	29	53	25	57
Age (y) median	69	69	69	72	63
B symptoms			66	11	
Splenomegaly (%)	73	82	81	11	42
Lymphadenopathy (%)	53	46	64^*^/28^**^	0	47
Hepatomegaly (%)	40	42	43	5	24
Skin lesions (%)	27	25	25	0	
Serous effusions (%)	14	21	19	0	
Other extra-nodal site (%)			13	0	
WBC (> 100 × 10^9^/L (%)	75	82	70	0	
WBC: 100 × 10^9^/L (median)			164	19	144
Hemoglobin < 10 g/dl (%)	36	55	15	0	
Hemoglobin: g/dl (median)					11.8
Platelets < 100 × 10^9^/l (%)	51	68	36	10	
Platelets: 100 × 10^9^/l(median)					85

^a^Aggressive forms, ^b^Indolent forms, ^*^Peripheral lymphadenopathy, ^**^ Abdominal or hilar lymphadenopathy, which will open the way for targeted therapies

### Differential diagnosis

Differential diagnosis of T-PLL often occurs with other mature T-cell leukemias including T-cell large granular lymphocytic leukemia (T-LGLL), Sézary syndrome (SS), and adult T-cell leukemia/lymphoma (ATLL). These disorders are characterized by heterogeneous clinical manifestations and outcome.

T-LGLL is an indolent clonal proliferation of cytotoxic T-cells. Patients are either asymptomatic, or present signs related to T-LGLL-induced cytopenia and associated with autoimmune disorders [51–53]. However, aggressive variants have been described with marked lymphocytosis, lymph-node infiltration, and hepatosplenomegaly [54–57]. T-LGLL cells in most cases have characteristic morphological features: large lymphocytes with abundant cytoplasm and fine or coarse azurophilic granules, which distinguish them from T-PLLs. However, in rare cases they do not display this typical morphology. In most cases, T-LGLL exhibits a constitutive mature post-thymic phenotype including CD3+, TCR αβ +, CD4-, CD5dim, CD7dim, CD8+, CD16+, CD27-, CD28-, CD45R0-, CD45RA+ and CD57+ characteristic of a constitutively activated T-cell phenotype [52, 53]. The less frequent CD3+CD56+ variant is associated with the most aggressive behavior [54–56]. In rare cases, T-LGLL exhibits a CD4+ phenotype with or without co-expression of CD8, associated with a significantly lower frequency of cytopenia, and of other associated autoimmune diseases [58, 59]. Cytogenetic abnormalities in T-LGLL have rarely been reported, in contrast to T-PLL, and there is no unique karyotypic abnormality [57, 60].

Sézary syndrome (SS) is an aggressive form of cutaneous T-cell lymphoma characterized by generalized erythroderma, peripheral blood involvement (frequently with PB eosinophilia) and generalized lymphadenopathy. The circulating neoplastic T-cells cells exhibit a distinct cerebriform morphology (and are called Sézary cells). The three main variants of SS are: primary SS (or Sézary leukemia) in which leukemia is present initially with erythroderma; SS arising in a setting of Mycosis Fungoides (MF); and SS that develops in the context of idiopathic erythroderma [61–63].

SS phenotypes are diverse but the tumor cells are typically T-helper memory lymphocytes, which are CD3+, CD4+ and CD5+. The neoplastic cells in SS express TCR αβ and have variable loss of expression of surface markers such as CD2, CD3, CD5, CD7 and CD26 [64–67]. In rare cases, SS cells express a CD8+ phenotype, and some exhibit a CD4-CD8- null phenotype [68]. Likewise, double CD4+ CD8+ is very rare, and should prompt a consideration of T-cell prolymphocytic leukemia [69].

Chromosomal abnormalities include gain of 17p11.2-q25.3 and 8q24.1-8q24.3 and loss of 17p13.2-p11.2; 10p12.1-q26.3, which are each observed in > 40% of SS. Duplication of 17q11.2 approximately q12 duplication is common to both MF and SS, which suggests that this is an early clonal event [70, 71] Gains of TCRB and TCRG, loss at least one copy of BCL2, gains of c-MYC and loss of c-MYC antagonists (MXI1and MNT) have also been reported [72–74].

Adult T-cell leukemia/lymphoma is a rare mature T-cell neoplasm associated with the retrovirus human T lymphotrophic virus type 1 (HTLV-1). Patients with aggressive ATL generally have a poor prognosis with a large tumor burden, hypercalcemia, frequent infectious complications due to profound T-cell immunodeficiency, and multi-organ failure. ATLL cells exhibits characteristic lymphocytes with a “flower cell” aspect, and express a mature T-cell phenotype: CD2+, CD3+, CD4+, CD8-, CD25+, CD7-, CD5+, CD25+ (strong and uniform), CD29+, CD45RO+, TCR αβ+, and HLA-DR+. There is aberrant loss of CD7 and CD26, and often downregulation of CD3 [75–77]. CD25 expression may be distinctive but not specific, because is also expressed in T-PLL and Sézary syndrome. ATLL with double-positive T cells (CD4+, CD8+) and only CD8+T lymphocytes have also been described [75–79].

Karyotypic abnormalities revealed by conventional cytogenetics or comparative genomic hybridization are complex, particularly in the acute forms, although there is no apparent characteristic abnormality; the abnormalities include aneuploidy (+3, +7, +21, −X, −Y), abnormalities of 3p, and translocations involving 14q11 and 14q32 sites of TCR(alpha) and TCR(delta) genes, respectively [76, 80, 81].

More sensitive array-comparative genomic hybridization showed that the lymphoma subtype is significantly more frequently associated with gains at 1q, 2p, 4q, 7p, and 7q and losses of 10p, 13q, 16q, and 18p, whereas the acute subtype showed a gain of 3/3p [82]. Differential diagnosis with T-PLL could occur in endemic area but rarely in the USA or Europe.

Several studies have suggested that some cases of mature T-leukemia may overlap morphologically and phenotypically with T-PLL. Pawson et al. reported nine cases of mature T cell disorder with morphological similarities to Sézary cells, but clinical and laboratory features were different from those seen in SS: lymphocytosis was between 12.7 to 133 × 10^9^, bone marrow infiltration, splenomegaly, lymphadenopathy, hepatomegaly, and CNS involvement in some cases; there was no skin involvement at diagnosis but this developed as a terminal event in two patients, one of whom presented a lymphoid infiltrate in the dermis, unlike the epidermotropism characteristic of SS. The cells from eight cases exhibit a mature T cell phenotype: TdT-, CD1a-, CD2+/-, CD3+, CD5+, CD25-. In three cases, the cells co-expressed CD4+ and CD8+, in two cases they were CD4-CD8+, in one case were CD4+CD8-, and in two cases the cells had a CD4-CD8- null phenotype. The CD7 antigen was expressed in six cases. One case was HTLV-1 serology positive and the diagnosis of adult T cell leukemia/lymphoma could not be excluded. Cytogenetic analysis of three cases revealed complex chromosomal abnormalities: in two cases, both isochromosome 8q and inversion 14(q11; q32) were found and in one case, t(X;14)(q28;q11) was detected. One patient showed complete remission of 21 months’ duration after treatment with Campath-1H. The median survival for the nine cases was 13 months. These observations suggest substantial similarity between Sézary cell leukemia or primary SS and some T-PLL cases. Cytogenetic investigations may help to distinguish them by identifying chromosomal abnormalities involving either TCL1 at 14q32.1, or MTCP1 at Xq28 in T-PLL [83].

Brito-Babapulle et al. explored the relationship between T leukemia with cerebriform nuclei and T-PLL. They performed a cytogenetic analysis with *in situ* hybridization of three cases with SS, and three cases with Sézary cell leukemia. Patients with SS had generalized erythroderma as expected, whereas patients with Sézary cell leukemia did not display apparent clinical skin involvement. All six cases had a mature T-cell phenotype, CD2+, CD3+, CD5+, and were CD1a-, CD11b-, CD16-, CD56-, CD57-. The three SS cases exhibited a CD4+CD8- phenotype, whereas all three Sézary cell leukemia cases co-expressed CD4 and CD8. All three cases with Sézary cell leukemia had inv (14)(q11;q32) and two had iso 8q, supporting the classification as a cerebriform variant of T-PLL. In contrast, SS cells did not show these abnormalities, but all three cases had iso (17q) or 17p+ abnormalities. One case of Sézary cell leukemia had iso (17q). These finding suggest that Sézary cell leukemia may be a variant of T-PLL rather than of SS [84].

Kussick et al. reported two cases of mature T cell lymphoproliferative disorder with cells exhibiting a CD3+, CD4+ phenotype, without clinical or morphological features suggesting either T-PLL (absence of the prolymphocytoid morphology, and of associated hepatosplenomegaly and/or generalized lymphadenopathy), or MF/SS, ATLL, T-LGLL or an underlying peripheral T cell lymphoma. Cytogenetic investigations were uninformative for diagnosis: findings were normal in one case and such tests not performed in the second case [85].

Herling et al. analyzed 102 cases of mature T-cell leukemia (38 T-PLL, 32 primary SS, 17 secondary SS, and 15 T-LGLL cases) and compared them to 10 cases of hepatosplenic lymphomas (HSTCL) that involved peripheral blood. The T-PLL cases showed rapidly rising PB lymphocyte counts with median peaks at 171.3 × 109/L; the corresponding values were while it was 5.9 × 10^9^/ L for in primary SS, 5.7 × 10^9^/L for secondary SS, and 3.6 × 10^9^/L for T-LGL leukemia cases. Some T-PLL cases had relatively low lymphocyte counts, and some primary SS and T-LGL leukemia cases had higher lymphocyte counts. T-PLL cases showed heterogeneous morphology, but SS cases exhibited the greatest variability including cells with large prolymphocytoid forms. CD4+CD8-, CD4+CD8+, CD4-CD8+, and CD4-CD8-phenotypes were found in 63%, 32%, 5%, and 0% of cases of T-PLL, respectively. CD4+CD8-, CD4+CD8+, CD4-CD8+, and CD4-CD8- phenotypes were observed in 94%, 3%, 0%, and 3% of primary SS cases, respectively, and in 13%, 0%, 87%, and 0% of T-LGL leukemia cases, respectively.

All cases of T-PLL and primary SS showed CD5 expression, whereas 75% of T-LGL leukemia cases were negative or only weakly positive for CD5. CD7 was expressed in 95% of tumor cells of T-PLL cases tested, but in only 61% of primary SS, 47% of secondary SS, 77% of T-LGL, and 67% of HSTCL of cases tested. Expression of CD26 was observed in 77% of T-PLL cases tested, but was less frequent in T-LGL (18%), primary SS (3%), secondary SS (18%), and HSTCL (0 of 3 tested) cases. TCR- γδ was expressed in 60% of HSTCL cases, only 14% of T-LGL leukemia cases, and no T-PLL or SS case.

TCL1 was expressed in 77% (10/13) of T-PLL cases tested by western blotting, and in 71% (27/38) of cases analyzed by immunostaining on paraffin sections; TCL1 expression was not detected in any primary SS, secondary SS, T-LGL, or HSTCL cases tested.

These findings reveal a degree of overlap between the features of T-PLL cases and those of other mature T-leukemias, especially primary SS. Nevertheless, some clinical and biological characteristics are more prevalent in some of these diseases than others. For example, rapidly rising and high peak PB lymphocyte counts, hepatosplenomegaly, presence of effusions and TCL1 expression are more frequently associated with T-PLL. Generalized erythroderma, PB eosinophilia, and lymphadenopathy are more typical of SS, and the presence of multiple cytopenias especially neutropenia and autoimmune disorders is most common in cases of T-LGLL [86].

More rarely, there is overlap with some HSCTL or other nodal lymphomas with PB involvement. Nevertheless, these diseases can generally be distinguished on the basis of TCR γδ rearrangement, pronounced hepatosplenomegaly and sinusoidal BM infiltration for HSCTL, or predominant nodal presentation and the pattern of lymph node infiltration for nodal lymphomas with PB involvement.

### Prognostic factors

In the series of the Royal Madsen Hospital, patients with serous effusions and hepatic or CNS involvement, or very bulky lymph node masses also had a poor response to therapy [87].

Age, gender, and race were not significant predictors of survival in a total of 272 cases of T-PLL studied between 1994–2010 based on multivariate analysis of the Surveillance Epidemiology and End Results (SEER-18) database. The median survival for the entire cohort was 21 months. A significant improvement in OS was noted for patients diagnosed after FDA approval of alemtuzumab (26 versus seven months) [88].

In a series from the MD Anderson Cancer Center, poor outcome was associated with age > 65 years, WBC over 40 × 10^9^/L at diagnosis, short lymphocyte doubling time, and high expression of the TCL-1 protein measured by flow cytometry and immunohistochemistry [20].

Bergmann et al reported that JAK3 mutations have a significant adverse impact on OS [41].

More recently, Aoki *et al.* reported that pleural effusion, a high absolute lymphocyte count, and a complex karyotype predicted an increased risk of death in 43 untreated patients among a series of 101 T-PLL cases [89].

However, these data are all derived from a small number of patients and do not allow us to determine the prognostic factors at diagnosis with certainty, or the indolent course of certain forms and their switch to the aggressive form.

### Therapeutic options

Alemtuzumab currently remains the first line treatment for T-PLL. It has improved the median OS obtained with alkylating agents or various purine analogues (Table 3). Despite a high response rate that can exceed 90% (Table 4), most patients who do not receive treatment intensification with allogeneic bone marrow transplantation will relapse with a median survival of 17-33 months in patients achieving a complete response (CR) [1, 20, 48–50, 87–92], increasing to 48 months after consolidation with autologous or allogeneic stem cell transplantation [93] (Table 5).

**Table 3:** Conventional chemotherapy in patients with T-PLL

	*N*	ORR (%)	CR (%)	Median OS (months)
alkylating agents (1)	32	31	3	7.5^a^
Pentostatin (90)^b^	55	45	9	17.5/9^c^
Pentostatin (91)^d^	25	8	0	4
Bendamustine (102)^b^	15	53.3	20	8.7

^a^ median OS of the entire group of 78 patients treated by various therapies, ^b^ relapsed/refractory and treatment-naïve patients, ^c^ median OS: 17.5 months for responders and 9 months for non-responders, ^d^ relapsed/refractory patients.

**Table 4:** Alemtuzumab-based therapy in patients with T-PLL

	*N*	ORR (%)	CR (%)	Median DOR (months)	Median DFS (months)	Median PFS^a^ (months)	Median OS (months)
Relapsed/refractory IV (98)	15	73	60				
Relapsed/refractory IV (87)	39	76	60		7		10
Relapsed/refractory IV (99)	76	51	39.5	8.7			7.5
Relapsed/refractory IV (100)	45	74	60			26% at 12 months	18% at 48 months
Subcutaneous first-line (100)	9	33	33			67% at 12 months	33% at 48 months
Intravenous first-line (100)	32	91	81			67% at 12 months	37% at 48 months
Intravenous in association with FCM (92)^b^	25	92	48			11.9	17.1
Intravenous in association with pentostatin (101)^c^	13	69	62			7.8	10.2

^a^ including SCT if applicable, ^b^ previously treated (n: 9) and treatment-naïve (n: 16), ^c^ previously treated (n: 8) and treatment-naïve (n: 5)

**Table 5:** Stem cell transplantation in patients with T-PLL

	*N*	CR rate before SCT (%)	CR rate after SCT (%)	TRM rate (%)	Relapse rate (%)	Median DFS (months)	Median PFS (months)	Median OS (months)
alemtuzumab+ auto-SCT ( 93)	15	86	100	7	60			52
alemtuzumab+allo-SCT (93)	13	69	92	31	33	24		33
Various therapy ^a^ +allo-SCT (109)	41	27		41% at 3 years	41% at 3 years	19% at 3 years		21% at 3 years
Various therapy +allo-SCT (110)	21						5.1	11.2 ^c^
Various therapy ^b^ +allo-SCT (111)	27	52	78	31% at 3 years	47		26% at 3 years	36% at 3 years

^a^ 24% of patients received alemtuzumab before allo-SCT, ^b^52% of patients received alemtuzumab before allo-SCT, ^c^ median OS for the entire cohort of 47 B and T-PLL

New treatment options are needed because of the high median age of most of the patients, associated comorbidities, and their impossibility to receive allogeneic bone marrow transplantation.

### Conventional chemotherapy

#### Alkylating agents and purine analogues

A study of conventional chemotherapy was performed on a large series of 78 patients who received various therapies including radiotherapy or no treatment. Of the 32 patients who received alkylating agents, nine (28%) had partial-responses (PRs). Five of 15 patients (33%) responded to CHOP, including one CR. Among the patients who received a regimen of the adenosine deaminase inhibitor, 2-deoxycoformycin (DFC, pentostatin), 31 were evaluable. Fifteen (48.4%) responded with three CRs and 12 PRs. The median survival for the entire group was 7.5 months. The median survival for patients treated with pentostatin (10 months) was better than for the remainder of the cohort (seven months), but was not statistically significant. Patients who responded to pentostatin had a median survival of 16 months, whereas that of non-responders was 10 months. The effect of hepatomegaly and age on survival was statistically significant [1].

The pentostatin regimen was tested in a series of 145 patients with post-thymic T-cell malignancies, among them 55 with T-PLL. Pentostatin was administered intravenously (IV), at 4 mg/m^2^/week for the first four weeks and then every two weeks until maximal response; the last 30 patients received weekly injections until maximal response. Among the patients, 67% had received previous systemic treatment. The ORR was 45% with CRs in 9% of cases. The median delay to remission was 6 months. Toxicity was low and pentostatin was generally well-tolerated. The median duration of survival from the start of therapy with pentostatin was longer in the responders (17.5 months) than in the non-responders (9 months) but did not reached statistical significance. No significant differences were observed when the results were analyzed according to previous treatment [90].

In a phase II trial, the EORTC group investigated the safety and efficacy of pentostatin in 92 cases of T lymphoid malignancies, including 25 evaluable T-PLL. All patients had relapsed/refractory disease: 76% had received ≤ 1 prior chemotherapy regimens and 24% had received ≥ 2. Pentostatin was administered at 4 mg/m^2^ every week for the first three weeks, then 4 mg/m^2^ every 14 days for another six weeks, followed by maintenance therapy. No CR was obtained and the PR rate was 8%. The disease-free survival rate and OS rates were 22 and 17.8 weeks, respectively [91].

Nelarabine is an arabinosylguanine nucleotide triphosphate (araGTP), a type of purine nucleoside analog, which inhibits DNA synthesis, causing cytotoxicity. Pre-clinical studies suggested that the drug was highly effective in immature T-cell diseases and, as expected, nelarabine showed significant cytotoxic activity against malignant T cell diseases in adult and pediatric populations [94, 95]. On the basis of the clinical results, nelarabine was approved by the FDA and the European Union for acute lymphoblastic leukemia and T-cell lymphoblastic lymphoma that has not responded to treatment or has relapsed following treatment with at least two chemotherapy regimens [96].

Nelarabine was evaluated in a phase I trial in 35 patients with progressive and advanced leukemia, including 11 with T-PLL, using three different protocols (consecutively for five days; on days 1, 3, and 5; or on days 1,3, and 5 in association with fludarabine). Responses were achieved in 20% (CR: 0%), 15% (CR: 5%), and 63% (CR: 13%) of patients, respectively. The histology, number of prior therapies, and fludarabine refractoriness status did not influence the response rate. Responses were durable, with a median time to treatment failure of 17 months in responders. The most common non-hematological toxicity was peripheral neuropathy in 21% of patients. Grade 4 neutropenia and thrombocytopenia occurred in 23% and 26% of patients, respectively [97].

### Alemtuzumab

Alemtuzumab is a humanized IgG1 kappa monoclonal antibody that binds to CD52, a protein present on the surface of mature lymphocytes, but not on the stem cells from which they are derived.

Alemtuzumab was first tested in a phase II trial in a series of 15 patients with T-PLL who had failed to achieve a CR with various previous chemotherapies, consisting mostly of pentostatin. Major responses occurred in 11 patients (73%) with nine (60%) CRs. Median survival from the time of treatment with alemtuzumab was eight months [98].

These preliminary results were confirmed in a study of 39 patients by Dearden *et al.* The patients received 30 mg Alemtuzumab IV, three times per week, until maximal response was achieved. All but two patients had received prior therapy, including 30 with pentostatin and none had achieved a CR. The ORR was 76% with 60% CR and 16% PR. Patients with serous effusions and hepatic or CNS involvement, or very bulky lymph node masses, responded poorly to therapy. The median DFS was seven months. The median OS for the entire group was 10 months. Survival was significantly longer in patients achieving CR (median, 16 months) relative to those with a PR (median, nine months) and non-responders (median, four months) [87].

The safety and efficacy of alemtuzumab (Campath-1H) was evaluated in a compassionate-use program in 76 previously treated (T-PLL) patients (including four chemotherapy-naive patients). Patients received 3, 10, and 30 mg alemtuzumab IV over the first three days, followed by 30 mg IV three times per week, for 4 to 12 weeks. The overall response rate was 51% with a 39.5% complete response rate (CRR). The median time to progression was 4.5 months, and median duration of CR 8.7 months. The median OS was 7.5 months. Ten patients experienced 15 infectious episodes during treatment (13%), leading to treatment discontinuation for three, and two treatment-related deaths [99].

In a pilot study (UKCLL05), nine previously untreated patients with T-PLL received 30 mg alemtuzumab subcutaneously, three times per week, until maximal response, for a maximum of 18 weeks. Three patients responded to treatment, all with a CR, for a CRR of 33%. Two patients (22%) died while on treatment. The outcome of these patients was compared with those of 32 treatment-naive patients with T-PLL who received alemtuzumab IV. These cases were managed similarly to those of the UKCLL05 patients. The ORR was 91%, with 81% CR. One half of the patients underwent stem cell transplantation after alemtuzumab therapy. The progression-free survival (PFS) at 12 months was 67% for both theIV and subcutaneously treated groups, with OS at 48 months of 37% and 33%, respectively. This finding demonstrated significantly lower response rates for subcutaneous administration of alemtuzumab relative to that administered IV as front-line therapy and the study was halted [100].

### Alemtuzumab + chemotherapy

A prospective multicenter phase II trial of the German CLL Study Group (GCLLSG) analyzed a combination chemotherapy of fludarabine , Mitoxantrone, and cyclophosphamide (FCM) followed by alemtuzumab consolidation in previously treated (*n* = 9) and treatment-naive (*n* = 16) patients with T-PLL. Patients received FMC, for up to four cycles, followed by alemtuzumab (A) consolidation, for up to 12 weeks. Of the 25 patients treated with FMC, 21 subsequently received alemtuzumab. The ORR to FMC was 68%, comprising six CR and 11 PR. Alemtuzumab consolidation increased the intent-to-treat ORR to 92% (12 CR; 11 PR). Median OS and PFS were 17.1 and 11.9 months, respectively. PFS tended to be shorter for patients with high-level TCL-1 expression. The most frequent grade 3/4 side effects under FMC-A were hematological toxicities. Among the 21 alemtuzumab-consolidated patients, 13 developed cytomegalovirus reactivations, of whom nine presented a clinically relevant infection [92].

Alemtuzumab was evaluated in a phase II trial in combination with pentostatin in 24 patients with a variety of T-cell leukemias and lymphomas. Thirteen patients had T-PLL, of whom eight had prior treatment with a median of two regimens. Patients received 30 mg alemtuzumab IV, three times weekly, for up to three months, and pentostatin at a dose of 4 mg/m^2^ IV weekly for four weeks, followed by alternate weekly administration for up to six months. Nine patients responded to treatment (eight CRs [CRR: 62%] and one PR), for an ORR of 69%. The median OS and PFS for patients with T-PLL were 10.2 and 7.8 months, respectively. The median event-free survival time for the responding patients with T-PLL was 20 months. Opportunistic infections, including cytomegalovirus reactivation, were common and there were five treatment-related deaths [101].

### Bendamustine

Bendamustine is a nitrogen mustard used in the treatment of chronic lymphocytic leukemia and indolent lymphomas. It displays a unique pattern of cytotoxicity, relative to conventional alkylating agents, and shows only partial cross-resistance with other DNA-binding anticancer agents and no cross-resistance with other alkylating agents.

Fifteen patients with T-PLL received bendamustine IV at a dose of 70–120 mg/m^2^ /d over 30–60 min, on days 1 and 2, every three weeks for an intended total of six cycles, with T-PLL. Seven patients received bendamustine after frontline alemtuzumab failure, two were treated after multiple therapies, and six were treated in the front line setting. Three patients (20%) achieved CR and five (33.3%) PR, for an ORR of 53.3%. The median PFS and OS were five and 8.7 months, respectively. PFS and OS did not correlate with alemtuzumab exposure or the line of treatment. Grade 3–4 neutropenia and thrombocytopenia were documented in three (20%) and two patients (13%), respectively [102].

### Stem-cell transplantation

Several case reports suggested a possible benefit from an immune graft-versus-leukemia (GVL) effect in T-PLL [103–108], leading several groups to investigate SCT in T-PLL. Several studies have suggested that allogeneic SCT may consolidate the response to initial chemotherapy and improve leukemic control and long-term survival in patients with T-PLL following induction treatment; however, it is associated with a significant rate of toxicity [93, 109–111].

SCT following alemtuzumab treatment was investigated in a multicentric series of 28 patients. Fifteen patients were consolidated with an autologous-SCT (median age: 58 years) and thirteen with an allogenic-SCT (median age: 51 years). Eleven patients were in their first CR, two in a second CR, and two in PR at the time of autologous-SCT. All 15 patients achieved CR following the autograft. Transplant-related mortality (TRM) was 7%. Nine patients (60%) relapsed at a median of 15 months. The median survival of the autograft patients was 52 months from the start of alemtuzumab treatment. At the time of allogenic-SCT, nine patients (69%) were in their first CR and four (31%) in PR. Twelve patients achieved a CR (92%). There were two cases of early and two of delayed TRM (31%), all for patients who received full-intensity conditioning. Four patients relapsed and died of progressive disease (31%), of whom all had sibling allografts. The median survival of the allograft patients was 33 months. All survivors had a matched unrelated donor. The median survival of all SCT patients was 48 months [57]. A retrospective control group of 23 non-transplanted patients (median age: 64 years) was selected based on achieved CR and survival > 6 months following alemtuzumab (in 39% of cases as first line). The median OS of the control group was only 20 months. Their five-year OS rate was 0%, whereas that of all transplanted patients was 34% [93].

The EBMT and Royal Marsden Consortium registry analyzed 41 T-PLL patients (median age: 51 years) that were allotransplanted in a salvage-like setting. The median time from diagnosis to treatment was 12 months. Eleven patients (27%) were in CR, twelve in PR (29%), thirteen had stable or progressive disease (32%), and the status of five was unknown (12%). Their lower pre-transplant CR rate was probably due, in part, to the small proportion of patients who received alemtuzumab (24%) prior to allogenic SCT. A total of 13 patients (31%) received reduced-intensity conditioning (RIC), and 65% of all myeloablative conditioning consisted of Total body irradiation (TBI). The three-year relapse-free survival (RFS) and OS were 19% and 21%, respectively, based on a median follow-up of surviving patients of 36 months. Multivariate analysis showed TBI and a < 12-month interval between diagnosis and HSCT to be associated with a favorable RFS. Three-year non-relapse-related mortality and relapse were each 41%, with most relapses occurring within the first year [109].

A multicenter CIBMTR registry (1995–2006) on 21 T-PLL (among 47 B- and T-PLL) patients after allogenic-SCT reported median PFS of 5.1 months at a short 13-month follow-up. The one-year OS for the entire cohort was 48% with median OS of 11.2 months. There were no significant differences in the one-year OS between patients treated with non-myeloablative or myeloablative transplant. There were also no significant differences based on sibling versus matched unrelated donor transplant, age, or whether the patient was in first CR or not [110].

The French society for stem cell transplantation (SFGM-TC) reported the outcome of 27 T-PLL cases. Prior to allogenic-SCT, 14 patients (52%) were in CR, 10 (37%) in PR, and three were refractory or had progressive disease (11%). The median time from diagnosis to HSCT was 8.5 months. Among the patients, 48% and 33% had received only one or two lines of pre-SCT treatments, respectively. Alemtuzumab constituted the most frequent pre-SCT therapy, (*n* = 14; all in first line setting). Following SCT, 21 patients (78%) achieved CR. Ten (37%) were still alive in CR based on a median follow-up for surviving patients of 33 months. The three-year PFS and OS were 26% and 36%, respectively. After SCT, 47% of patients relapsed at a median of 11.7 months. The overall cumulative incidence of TRM was 31% after three years [111].

Allogenic SCT may improve OS in patients with T-PLL after induction by alemtuzumab, especially for those in first CR and allotransplanted in the first year after diagnosis. A reduced conditioning regimen can help reduce TRM, currently from 30–40%. The relapse rate is still significant at 30–40% and improvement of the response to induction therapy is an important option. The best treatment for relapse remains to be found, but may include donor lymphocyte infusions in allotransplanted patients aided by monitoring of minimal residual disease by flow cytometry or PCR or NGS [112].

However, the diverse situations and outcomes and ongoing search for more effective regimens, as recently reported by Herling, highlight the need for extensive optimization of the allo-SCT procedures and the resolution of several issues. These include reducing TRM, pausing the administration of pre-SCT alemtuzumab for sustained engraftment, and identifying predictors that involve the success of conditioning [113].

Otherwise, only 30-50% of patients with T-PLL can benefit from allogenic SCT. Autologous SCT may be another option for patients who are in generally good condition and aged 65 years or less.

#### New approaches

T-PLL is an aggressive disease with a poor outcome that needs new therapeutic approaches. After alemtuzumab treatment, most patients rapidly relapse. Allotransplanted patients have a longer median survival, but allogenic-SCT is only suitable for a minority of patients.

Cytogenetic and molecular analysis shows that T-PLL exhibits substantial mutational activation of the TCL-1-AKT and MTCP1 proto-oncogenes, the IL2RG-JAK1-JAK3-STAT5B axis, or somatic mutations in genes involved in DNA binding and chromatin remodeling, or epigenetic regulation, that could be candidates for targeted therapies.

#### AKT inhibitors

The primary event in T-PLL is the inv(14)/t(14;14) or t(X;14), leading to activation of TCL1A or MTCP1, respectively. Both proteins interact with the AKT kinase and activate the AKT pathway. Thus, novel agents that target this pathway could be a treatment option in T-PLL.

The preclinical efficacy of the AKT inhibitor, MK-2206, was explored on leukemic T-cells isolated from T-PLL patients. The AKT inhibitor, MK-2206 was able to induce dose-dependent apoptosis of isolated PBMCs (containing > 90% leukemic T-cells) of T-PLL patients (*n* = 4) (IC50: 5 µM) [15].

Recently, the AKT inhibitor MK-2206 was evaluated in a phase II trial in 59 patients with relapsed or refractory lymphoma. It was administered orally at 200 mg once weekly in 28-day cycles, for up to 12 cycles, in the absence of progression or significant toxicity. The dose was adjusted, based on tolerance, and finally, 33 patients received 300 mg, two 250 mg, 16 200mg, and eight 135 mg. Eight patients experienced an objective response (two CR and six PR, overall response rate 14%), with a median duration of response of 5.8 months. The median duration of EFS in all patients was 2.8 months. The most common toxicity observed in this study was rash (any Grade 53%, Grade 3 15%). These promising results show that MK-2206 could be a potential therapeutic agent for T-PLL patients [114].

#### Epigenetic therapy

EZH2 is the catalytic subunit of the polycomb repressive complex 2 (PRC2) and is responsible for tri-methylation of histone 3 lysine 27 (H3K27), a mark of transcriptional repression. Recurrent gain-of-function mutations and overexpression of EZH2 drive and promote malignant transformation, such as B-cell lymphomagenesis [115–117]. EZH2 also plays a role in T-cell differentiation and mutations of EZHZ have been found in various T-cell malignancies. Several epigenetic mutations were reported in T-PLL, including a frameshift mutation in the SET domain of EZH2, providing a rationale for inhibiting EZH2 as a potential anti T-PLL strategy [4].

#### Anti EZH2 inhibitor-GSK2816126

GSK2816126 is a highly selective and potent inhibitor of both wild type and mutant EZH2, decreases H3K27 tri-methylation, releases transcriptional repression of PRC2 target genes, and induces anti-proliferative activity in several wild type and mutant EZH2 cancer cell lines. GSK2816126 was investigated in a combined phase I accelerated plus classic 3+3 study, with adaptive Bayesian design dose escalation, in 30 patients with relapsed/refractory lymphomas, solid tumors, and multiple myeloma. Patients received two IV doses weekly for 21 days (3 weeks-on/1 week-off) in a 28-day cycle. GSK2816126 was well tolerated with no DLTs observed. Dose expansion is ongoing at 3,000 mg given IV twice weekly (21 days/28 days). The most frequent drug-related adverse events reported were fatigue (53%), nausea (30%), anemia (20%), and vomiting (20%). Of 22 evaluable patients, one, an advanced GCB + DLBCL patient, had a confirmed durable PR. Seven patients had stable disease, including an advanced FL patient with 45% tumor regression and a cholangiocarcinoma patient with stable disease lasting > 6 cycles [118].

#### JAK inhibitors

Mutations in the JAK-STAT pathway occur frequently, especially JAK3 mutations, which have been reported in 21-42% of T-PLL, suggesting that T-PLL patients could benefit from JAK inhibitors, mostly JAK3 inhibitors.

Several early studies reported reduced cell growth, increased apoptosis, and reduced survival following treatment with JAK3 inhibitors in different experimental systems expressing the above-mentioned activating JAK3 mutations [119–122]. These results provided proof-of-principle evidence that JAK3 inhibitors could provide a therapeutic benefit to patients with hematological malignancies carrying activating JAK3 mutations.

However, several issues remain to be resolved, because some patients have more than one JAK mutation and the coexistence of JAK1 and JAK3 [40], which would lead to treatment resistance with either JAK1 or JAK3 kinase inhibitors. Indeed, JAK inhibitors are confronted with eventual treatment resistance, as are other tyrosine kinase inhibitors that target one specific part of the enzyme, typically the ATP-binding pocket; the mutation of a single amino acid can result in resistance of the oncogenic kinase. Furthermore, a new mechanism of acquisition of resistance to JAK inhibitors was recently reported. Cells transformed by a JAK3 mutation became resistant to a JAK3-selective inhibitor by acquiring another activating mutation in JAK1, whereas cells that originally had a JAK1-activating mutation became resistant to inhibitors by acquiring another activating mutation in JAK3, highlighting the cooperation between JAK1 and JAK3 mutants in T-cell transformation [123]. The therapeutic combination of JAK3- and JAK1-selective inhibitors could be an option, as they have been shown to act synergistically on the growth of JAK3 mutant cells in a T-ALL mouse model [124]. Finally, JAK inhibitors could be used in combination with current therapies.

#### Tofacinib

Tofacitinib (CP-690,550) is an oral, small-molecule JAK inhibitor, FDA-USA-approved in 2012 for the treatment of rheumatoid arthritis, that demonstrated high selectivity for JAK3 inhibition with an enzyme inhibitory potency of 1 nmol/L and showed 20- to 100-fold less selectivity for JAK2 and JAK1, respectively [125].

CP-690,550 was tested in a human NKCL xenograft mouse model, and deactivate the dissemination of MEC04 NKCL cells to the bone marrow, blood, and spleen. CP-690,550 could be hindered cell proliferation, viability, metastatic dissemination, and survival of malignant cells in NKCL [122].

#### Ruxolitinib

Ruxolitinib is a USA-FDA approved selective JAK1/2 inhibitor for the treatment of myeloproliferative neoplasms, including intermediate or high risk myelofibrosis and polycythemia vera in patients who did not respond well to, or did not tolerate, hydroxyurea.

Recent evidence suggests that mutations in the JAK/STAT pathway may play an essential role in the pathogenesis of cutaneous T-cell lymphoma (CTCL). Perez *et al.* detected somatic mutations in either JAK1 or JAK3 genes in up to 7 patients and 1 cell line among a total of forty-six patients and two cell lines, most located in the pseudokinase domain. They then analyzed the biological effects of the specific inhibition of JAK/STAT signaling in human CTCL cell lines, including HuT-78 cells carrying mutated JAK1 and JAK3 genes, by incubating them with increasing doses of ruxolitinib (INCB018424). The cells exhibited activated basal STAT phosphorylation in the absence of serum, possibly due to multiple activating mechanisms including, but not restricted to, JAK mutations. They showed dose-dependent inhibition of cell proliferation and rapid inhibition of STAT activation, including in the HuT-78 cell line. In contrast, they reported the induction of only moderate cytotoxicity by JAK inhibition, suggesting that inhibition of the JAK/STAT pathway does not simply have a direct cytotoxic effect, but efficiently prevents malignant cell growth in CTCL through other mechanisms as well. [126]. Ruxolitinib is being studied in relapsed B-cell NHL and PTCL (NCT01431209).

Several other agents are currently being evaluated for various indications and could be tested in T-PLL: decernotinib (VX-509), a selective oral JAK3 inhibitor, which showed 25–120-fold selectivity for JAK3 over other JAKs (JAK1, JAK2, and TYK2) based on similar cell-based assays [127], peficitinib (ASP015K), an oral JAK1/JAK3 inhibitor, which also inhibited IL-2-dependent T cell proliferation *in vitro* and STAT5 phosphorylation *in vitro* and *ex vivo* [128], and filgotinib (GLPG0634), a selective oral JAK1 inhibitor [129].

The challenge for these second-generation inhibitors will be to show better specific selectivity within the JAK family with a more manageable safety profile and a better prognosis during long-term/chronic usage.

#### STAT5 inhibitors

The selective STAT5 inhibitor, pimozide, which is active in myeloid leukemia, induces a specific and profound reduction in cell proliferation, viability, and pSTAT5 levels of STAT5-activated cells (HUT78 cells), as well as primary T-PLL cells [4], and could also be another targeted treatment.

#### PARP inhibitors

The concept of synthetic lethality consists of the simultaneous inactivation of two genes to causes cell death, whereas cells remain viable following inactivation of either of the two genes alone. Poly (ADP-ribose) polymerase (PARP) inhibition requires DNA double strand break repair and should selectively sensitize ATM-dysfunctional cells to die, by inducing conversion of one form of DNA damage into another. The sensitivity to the poly (ADP-ribose) polymerase inhibitor olaparib (AZD2281) was investigated *in vitro* in ATM mutant lymphoid tumors. Pharmacologic inhibition of tumor cell growth was ATM-dependent and was mediated through mitotic catastrophe, independently of apoptosis. Olaparib treatment also sensitized ATM-null tumor cells to DNA-damaging agents [130].

#### Immunotherapy combined with epigenetic therapy

A recent study consisted of adding 2CDA-cladribine and anti-HDAC inhibitors to the monoclonal antibody-anti CD52-alemtuzumab. The rationale of the study was based on the ability of 2CDA-cadribine, a purine analogue with known hypomethylating activity to synergistically increase the expression of silenced tumor suppressors, when combined with anti-HDAC inhibitors, promoting cell death. Nine patients with T-PLL were treated with alemtuzumab and cladribine, with or without an HDAC inhibitor (vorinostat or valproic acid or romidepsine), under various conditions (refractory or relapse, or initial presentation). Eight patients achieved CR and one PR. One patient received an allogeneic transplant and remains in complete remission. The others all relapsed. Retreatment of the patients after relapse was effective, and the administration of epigenetic agents overcame initial alemtuzumab resistance. The authors hypothesized that epigenetic drugs induce increases in antibody-dependent cell mediated cytotoxicity (ADCC) [131].

The same group showed the induction of CD30 after epigenetic therapy in T-PLL cells by proteomic and transcriptomic analysis, but the mechanism by which epigenetic agents induce the expression of CD30 is not completely understood. In this context, a patient in the fifth line setting presenting with refractory skin lesions, was treated with the antibody drug conjugate, brentuximab vedotin. A biopsy showed lymphocytes testing weakly positive for CD30 surface expression infiltrating the dermis. The patient achieved CR but relapsed five months later with systemic disease [132].

## CONCLUSIONS

T-PLL is a rare disease with a poor prognosis. Flow cytometry has led to better characterization of the cells involved in this disease and cytogenetics and molecular biology have revealed much about its pathophysiology. The genetic causes and oncogenic signaling events responsible for the malignant transformation of T-PLL are yet to be discovered.

T-PLL has a particular genomic and transcriptomic profile with a complex karyotype and recurrent chromosomal abnormalities of several genes involved in cell cycle regulation, apoptosis, DNA repair, and epigenetic modulation [3, 17–19, 22–28, 30, 31]

Conventional treatment has provided disappointing results. Treatment with alemtuzumab significantly improves survival relative to other agents used to treat T-PLL. IV administration should be the preferred route. Alemtuzumab in combination with pentostatin or another purine analogue should be considered for patients not responding to alemtuzumab, mostly those with very aggressive tumor forms or extramedullary localization. Nevertheless, the response to alemtuzumab is often transient and consolidation by SCT should be discussed with all patients who achieve complete remission and who are otherwise eligible. However, this procedure is reserved only for the minority of patients in good general condition.

Prospective trials to define the most effective therapeutic strategy and improve clinical responses to frontline therapy are necessary. New approaches using well-tolerated targeting therapies involving growth and survival signals are needed for the majority of patients unable to receive intensive chemotherapy.
